# Supplementary material for: Position-dependent function of human sequence-specific transcription factors
Source: Nature. 2024 Jul 17;631(8022):891–8. doi: 10.1038/s41586-024-07662-z (PMC11269187; doi:10.1038/s41586-024-07662-z)
Supplement: Supplementary file 1 — This file contains Supplementary Figs 1–7 and Supplementary References. [file 41586_2024_7662_MOESM1_ESM.pdf]

nature portfolio

https://doi.org/10.1038/s41586-024-07662-z

Supplementary information

Position-dependent function of human sequence-specific transcription factors

In the format provided by the authors and unedited

Position-dependent function of human sequence-specific transcription factors

Sascha H. Duttke[1*], Carlos Guzman[2], Max Chang[2], Nathaniel P. Delos Santos[2], Bayley R. McDonald[1], Jialei Xie[3], Aaron F. Carlin[3], Sven Heinz[2*] & Christopher Benner[2*]

1. School of Molecular Biosciences, College of Veterinary Medicine, Washington State University, Pullman, WA, USA.
2. Department of Medicine, Division of Endocrinology, U.C. San Diego School of Medicine, La Jolla, California, 92093 USA.
3. Department of Pathology and Medicine, U.C. San Diego School of Medicine, La Jolla, California, 92093 USA.

*Correspondence: sascha.duttke@wsu.edu (S.H.D), sheinz@health.ucsd.edu (S.H), cbenner@health.ucsd.edu (C.B.)

Supplementary Information

Supplementary Tables 3
Supplementary Figure 1 4
Supplementary Figure 2 5
Supplementary Figure 3 7
Supplementary Figure 4 11
Supplementary Figure 5 13
Supplementary Figure 6 10
Supplementary References 14

Supplementary Tables

Supplementary Table S1: List of experiments performed in this study available in GSE199431. Note that additional data utilized in the study, including K562 csRNA-seq and C57Bl/6 mouse macrophage csRNA-seq experiments, were previously published and available from GSE135498.

Supplementary Table S2: List of key sequences used in the creation of TSS-MPRA DNA inserts. Full sequence lists are available as supplementary files in FASTA format from GSE199431.

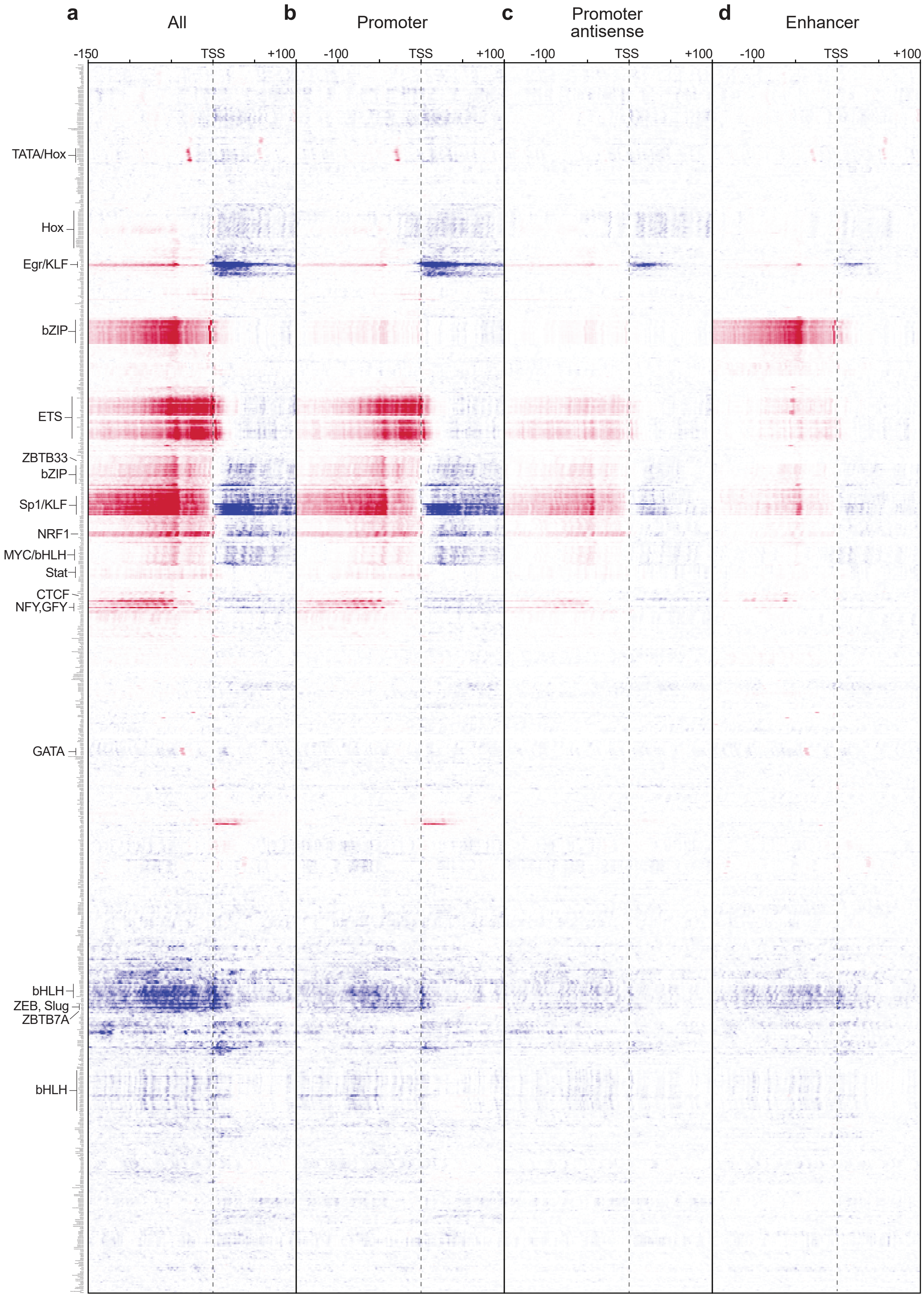

Supplementary Figure 1. Positional preferences of TF binding sites relative to TSSs across different types of regulatory elements: Overview of the enrichment or depletion of all 463 known human TF binding sites relative to the TSS for **a**, all TSRs (identical to Fig. 1d), **b**, promoters, **c**, loci initiating upstream antisense transcription, and **d**, putative enhancers.

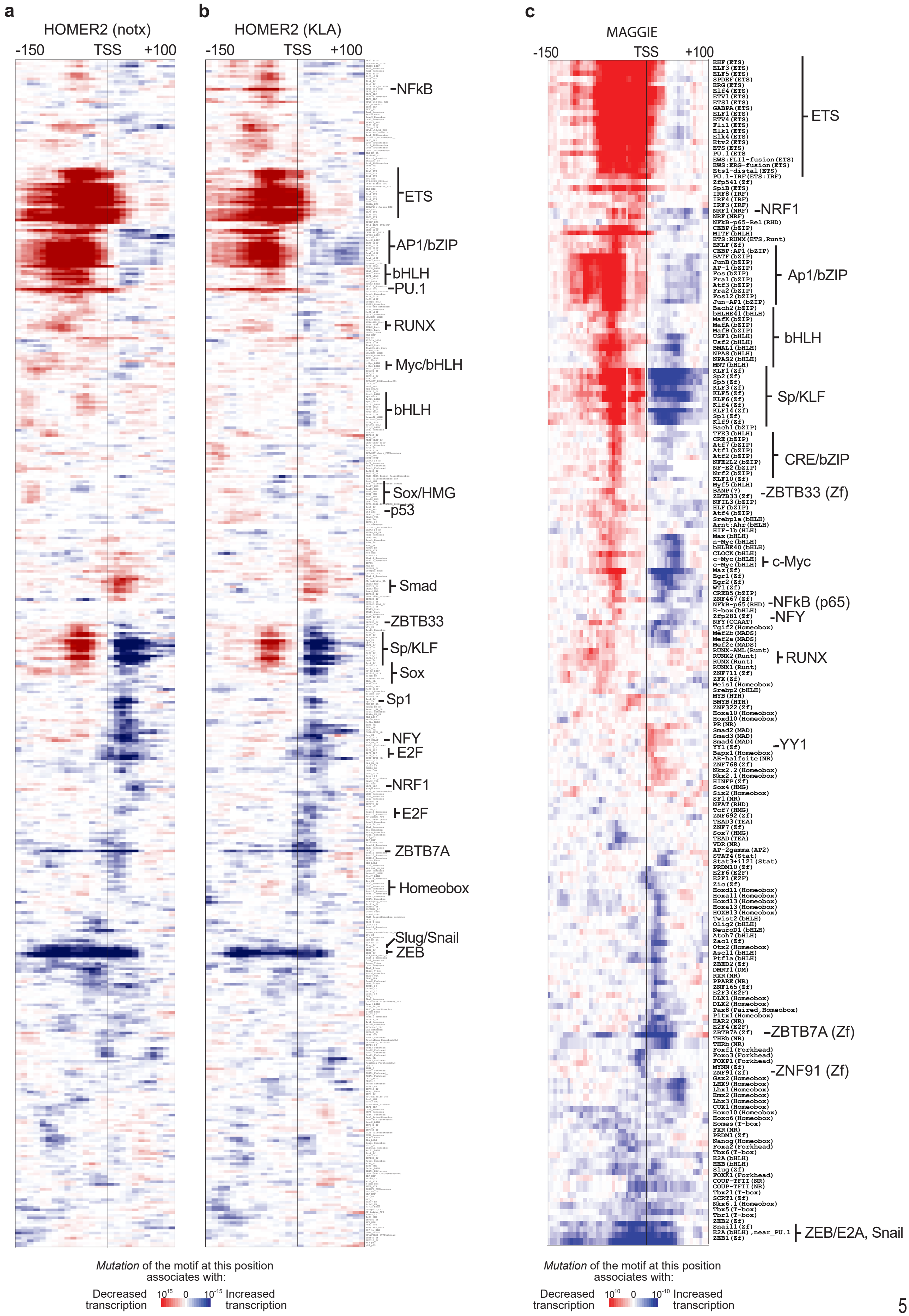
a
HOMER2 (notx)
-150
TSS
+100
b
HOMER2 (KLA)
-150
TSS
+100
NFkB
ETS
AP1/bZIP
bHLH
PU.1
RUNX
Myc/bHLH
bHLH
Sox/HMG
p53
Smad
ZBTB33
Sp/KLF
Sox
Sp1
NFY
E2F
NRF1
E2F
ZBTB7A
Homeobox
Slug/Snail
ZEB
c
MAGGIE
-150
TSS
+100
EHF(ETS)
ELF3(ETS)
ELF5(ETS)
SPDEF(ETS)
ERG(ETS)
Elf4(ETS)
ETV1(ETS)
ETS1(ETS)
GABPA(ETS)
ELF1(ETS)
ETV4(ETS)
Fli1(ETS)
Elk1(ETS)
Elk4(ETS)
Etv2(ETS)
ETS(ETS)
PU.1(ETS)
EWS:FLI1-fusion(ETS)
EWS:ERG-fusion(ETS)
Ets1-distal(ETS)
PU.1-IRF(ETS:IRF)
Zfp541(Zf)
SpiB(ETS)
IRF8(IRF)
IRF4(IRF)
IRF3(IRF)
NRF1(NRF)
NRF(NRF)
NFkB-p65-Rel(RHD)
CEBP(bZIP)
MITF(bHLH)
ETS:RUNX(ETS,Runt)
EKLF(Zf)
CEBP:AP1(bZIP)
BATF(bZIP)
JunB(bZIP)
AP-1(bZIP)
Fos(bZIP)
Fra1(bZIP)
Atf3(bZIP)
Fra2(bZIP)
Fosl2(bZIP)
Jun-AP1(bZIP)
Bach2(bZIP)
bHLHE41(bHLH)
MafK(bZIP)
MafA(bZIP)
MafB(bZIP)
USF1(bHLH)
Usf2(bHLH)
BMAL1(bHLH)
NPAS(bHLH)
NPAS2(bHLH)
MNT(bHLH)
KLF1(Zf)
Sp2(Zf)
Sp5(Zf)
KLF3(Zf)
KLF5(Zf)
KLF6(Zf)
Klf4(Zf)
KLF14(Zf)
Sp1(Zf)
Klf9(Zf)
Bach1(bZIP)
TFE3(bHLH)
CRE(bZIP)
Atf7(bZIP)
Atf1(bZIP)
Atf2(bZIP)
NFE2L2(bZIP)
NF-E2(bZIP)
Nrf2(bZIP)
KLF10(Zf)
Myf5(bHLH)
BANP(?)
ZBTB33(Zf)
NFIL3(bZIP)
HLF(bZIP)
Atf4(bZIP)
Srebp1a(bHLH)
Arnt:Ahr(bHLH)
HIF-1b(HLH)
Max(bHLH)
n-Myc(bHLH)
bHLHE40(bHLH)
CLOCK(bHLH)
c-Myc(bHLH)
c-Myc(bHLH)
Maz(Zf)
Egr1(Zf)
Egr2(Zf)
WT1(Zf)
CREB5(bZIP)
ZNF467(Zf)
NFkB-p65(RHD)
E-box(bHLH)
Zfp281(Zf)
NFY(CCAAT)
Tgif2(Homeobox)
Mef2b(MADS)
Mef2a(MADS)
Mef2c(MADS)
RUNX-AML(Runt)
RUNX2(Runt)
RUNX(Runt)
RUNX1(Runt)
ZNF711(Zf)
ZFX(Zf)
Meis1(Homeobox)
Srebp2(bHLH)
MYB(HTH)
BMYB(HTH)
ZNF322(Zf)
Hoxa10(Homeobox)
Hoxd10(Homeobox)
PR(NR)
Smad2(MAD)
Smad3(MAD)
Smad4(MAD)
YY1(Zf)
Bapx1(Homeobox)
AR-halfsite(NR)
ZNF768(Zf)
Nkx2.2(Homeobox)
Nkx2.1(Homeobox)
HINFP(Zf)
Sox4(HMG)
Six2(Homeobox)
SF1(NR)
NFAT(RHD)
Tcf7(HMG)
ZNF692(Zf)
TEAD3(TEA)
ZNF7(Zf)
Sox7(HMG)
TEAD(TEA)
VDR(NR)
AP-2gamma(AP2)
STAT4(Stat)
Stat3+il21(Stat)
PRDM10(Zf)
E2F6(E2F)
E2F1(E2F)
Zic(Zf)
Hoxd11(Homeobox)
Hoxa11(Homeobox)
Hoxd13(Homeobox)
Hoxa13(Homeobox)
HOXB13(Homeobox)
Twist2(bHLH)
Olig2(bHLH)
NeuroD1(bHLH)
Atoh7(bHLH)
Zac1(Zf)
Otx2(Homeobox)
Ascl1(bHLH)
Ptf1a(bHLH)
ZBED2(Zf)
DMRT1(DM)
RXR(NR)
PPARE(NR)
ZNF165(Zf)
E2F3(E2F)
DLX1(Homeobox)
DLX2(Homeobox)
Pax8(Paired,Homeobox)
Pitx1(Homeobox)
EAR2(NR)
E2F4(E2F)
ZBTB7A(Zf)
THRb(NR)
THRb(NR)
Foxf1(Forkhead)
Foxo3(Forkhead)
FOXP1(Forkhead)
MYNN(Zf)
ZNF91(Zf)
Gsx2(Homeobox)
LHX9(Homeobox)
Lhx1(Homeobox)
Emx2(Homeobox)
Lhx3(Homeobox)
CUX1(Homeobox)
Hoxc10(Homeobox)
Hoxc6(Homeobox)
Eomes(T-box)
FXR(NR)
PRDM1(Zf)
Nanog(Homeobox)
Foxa2(Forkhead)
Tbx6(T-box)
E2A(bHLH)
HEB(bHLH)
Slug(Zf)
FOXK1(Forkhead)
COUP-TFII(NR)
COUP-TFII(NR)
Tbx21(T-box)
SCRT1(Zf)
Nkx6.1(Homeobox)
Tbx5(T-box)
Tbr1(T-box)
ZEB2(Zf)
Snail1(Zf)
E2A(bHLH),near_PU.1
ZEB1(Zf)
ETS
NRF1
Ap1/bZIP
bHLH
Sp/KLF
CRE/bZIP
ZBTB33 (Zf)
c-Myc
NFkB (p65)
NFY
RUNX
YY1
ZBTB7A (Zf)
ZNF91 (Zf)
ZEB/E2A, Snail
Mutation of the motif at this position associates with:
Decreased transcription
10^15
0
10^-15
Increased transcription
Mutation of the motif at this position associates with:
Decreased transcription
10^10
0
10^-10
Increased transcription

Supplementary Figure 2. Robustness of position-dependent function of TF binding sites measured by analyzing naturally occurring genetic variation between two strains of mice. Comparison of methods as well as activated and rested bone marrow-derived macrophages. **a**, Overview heatmap all 463 known human TF motifs where mutations that alter TF binding site strength significantly associated with changes in initiation strength in a distance-dependent manner relative to the TSSs of untreated bone marrow-derived macrophages (BMDMs) [1], as calculated using HOMER2 (Duplicate to Fig. 2f). Note, not all TFs are expressed in BMDMs. **b**, Position-dependent function of TF binding sites as calculated in Fig. 2f/Supplementary Figure 3a) but for BMDMs treated with infection-mimicking KLA (one increase in ETS/AP1, NFkB and ISRE). **c**, Position-dependent function of TF binding sites in BMDMs (notx) as calculated using MAGGIE [2] (please see methods). Annotated overview heatmap of 207 of the 463 HOMER2 known TF motifs where mutation of the TF binding sites significantly associated with changes in transcription as measured by csRNA-seq in untreated bone marrow-derived macrophages. Note: while the analysis using HOMER 2 was limited to single nucleotide variants to enable the correction of position-dependent variant bias (Extended Data Fig. 7a,b), MAGGIE also uses indels and structural variation, increasing the sensitivity of the analysis, but disregarding position-dependent or variant bias.

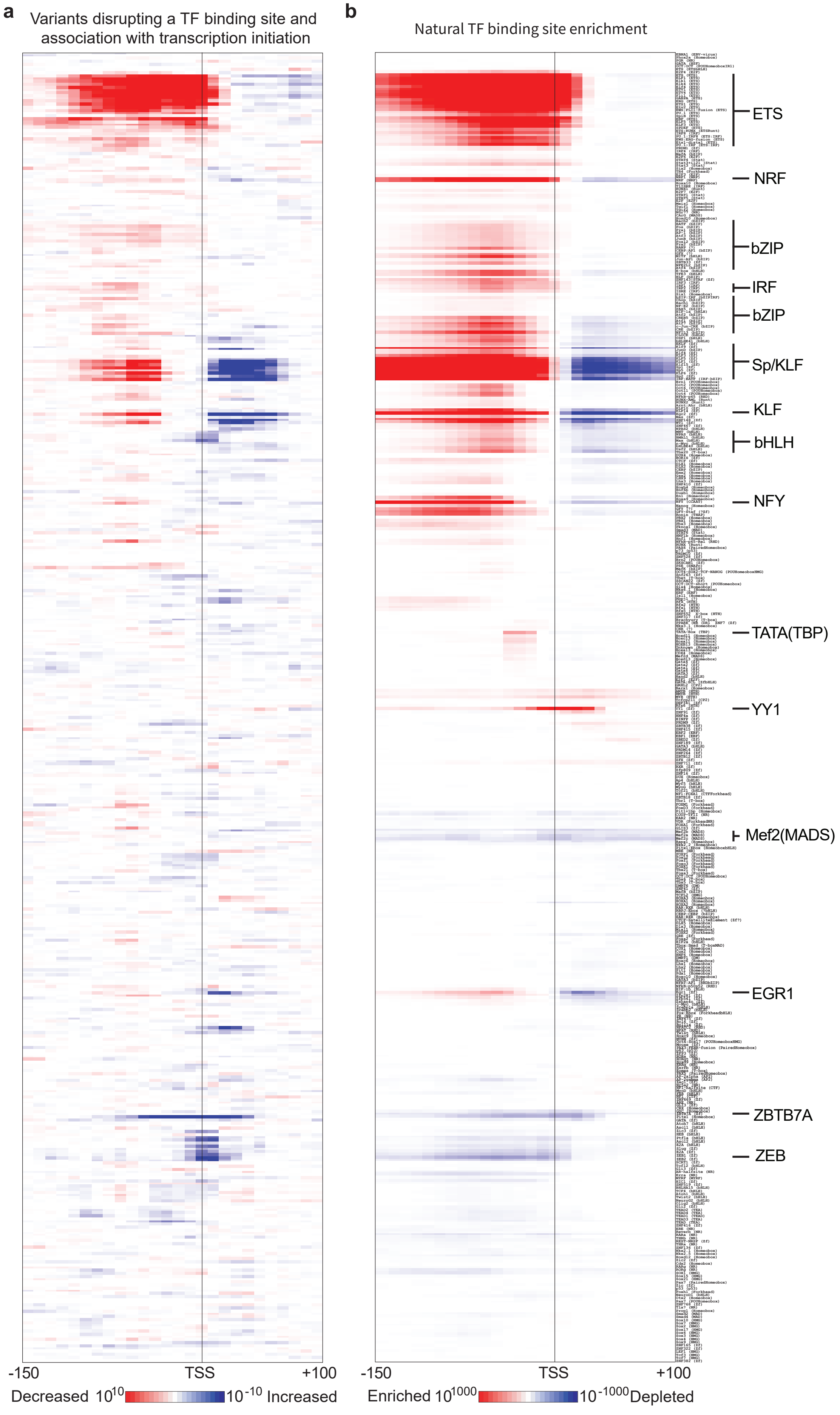
a
Variants disrupting a TF binding site and association with transcription initiation
-150
TSS
+100
Decreased 10^{10}
10^{-10} Increased
b
Natural TF binding site enrichment
ETS
NRF
bZIP
IRF
bZIP
Sp/KLF
KLF
bHLH
NFY
TATA(TBP)
YY1
Mef2(MADS)
EGR1
ZBTB7A
ZEB
-150
TSS
+100
Enriched 10^{1000}
10^{-1000} Depleted

Supplementary Figure 3. Positional TF binding site enrichment and position-dependent activity of TFs based on the analysis of genetic variants and TSS activity across 67 human individuals: Detailed figure for Fig. 5a. **a**, Overview heatmap of all 463 human HOMER2 TF motifs where mutations that alter TF binding site strength are associated with changes in initiation strength in a distance-dependent manner relative to the TSS. **b**, Relative enrichment of TF bindings site relative to the TSSs. Note, not all TFs are expressed.

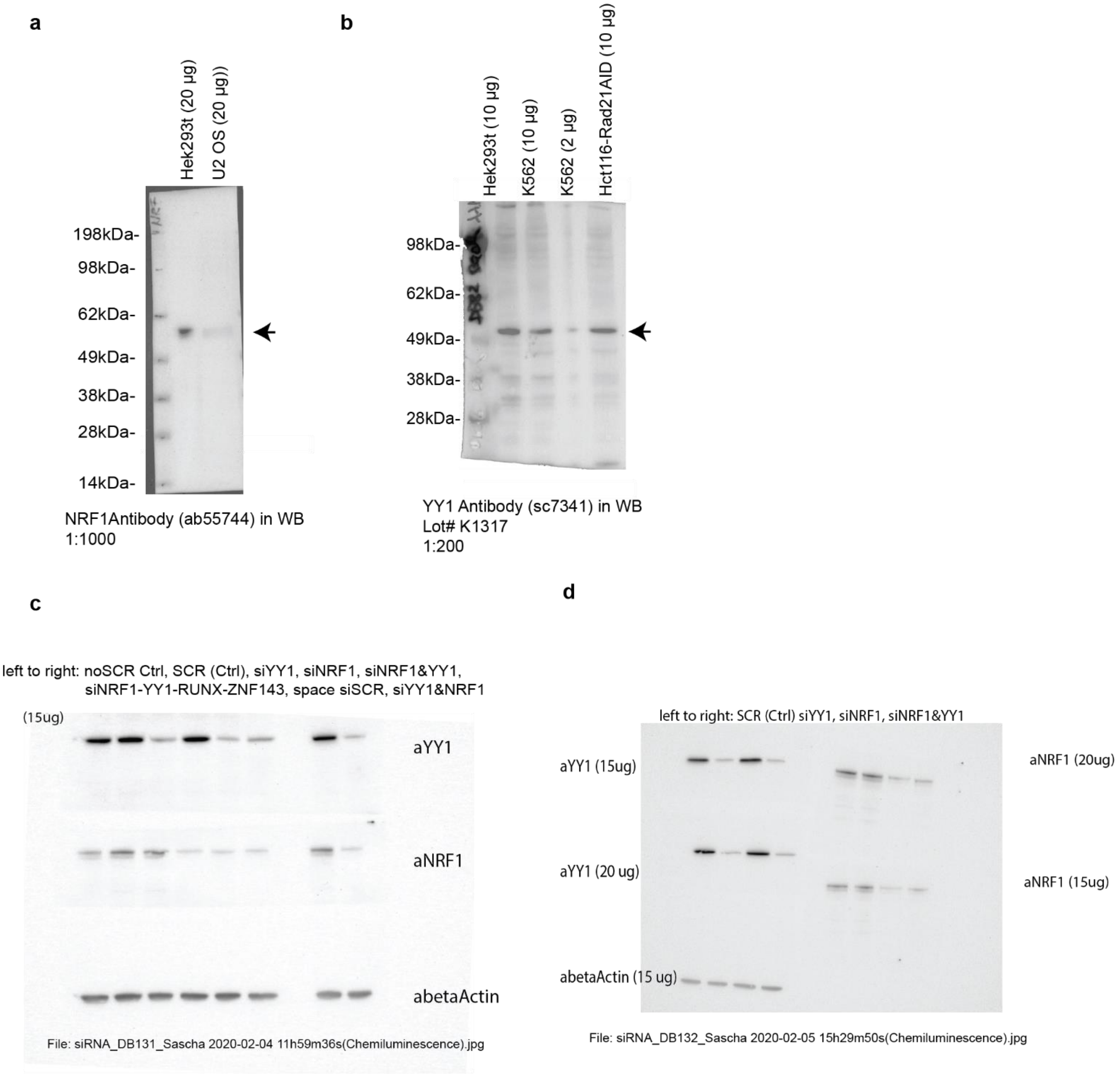

Supplementary Figure 4. Western blots. **a**, NRF1 and **b**, YY1 antibody testing. **c**,**d**, western blot quantification for siRNA experiments used in this paper. Note: csRNA-seq experiments for siYY1&NRF1, siNRF1-YY1-RUNX-ZNF143 were not used in this paper

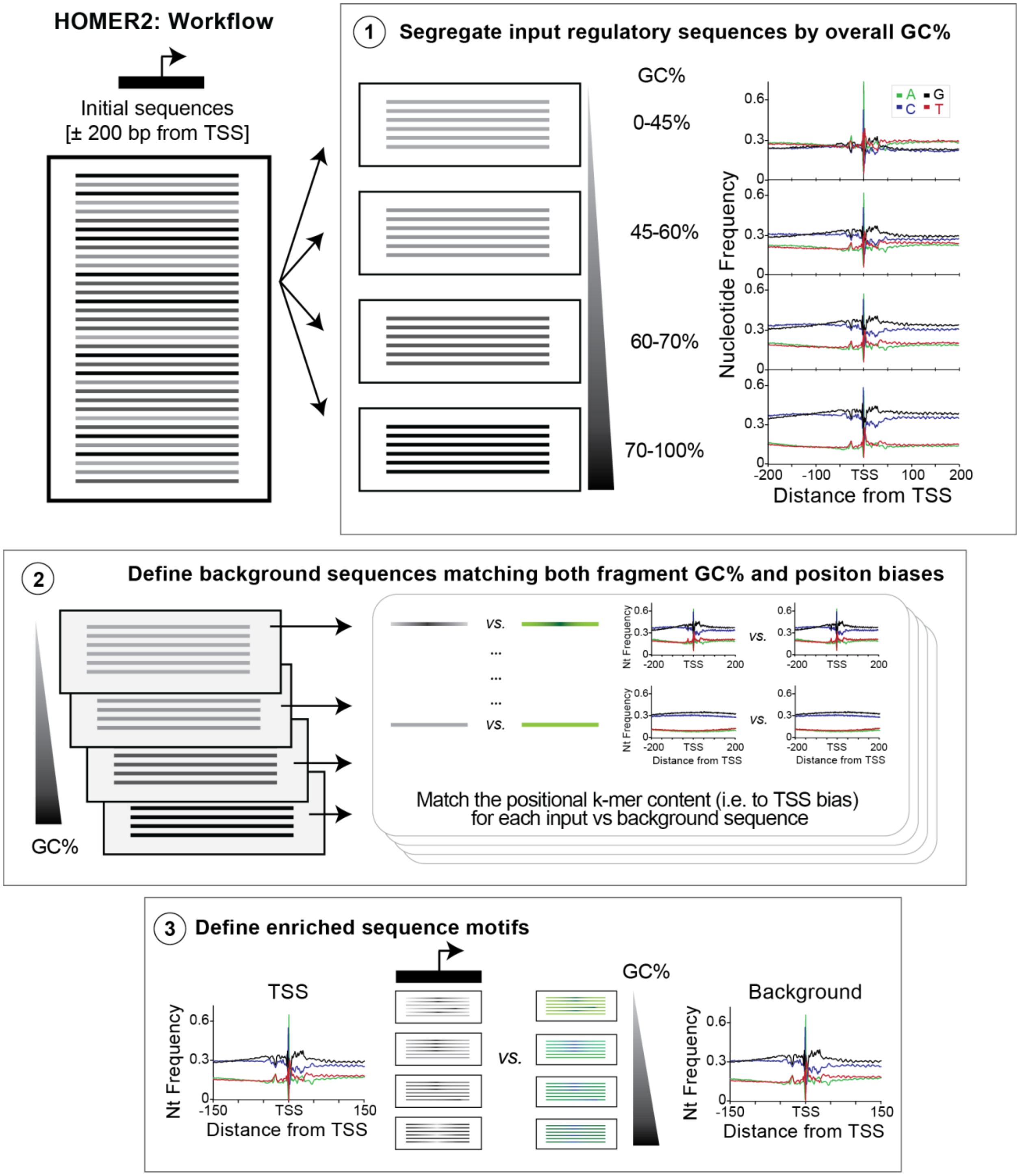

Supplementary Figure 5. HOMER2 workflow: Input sequences are first binned based on total GC content. Next, background sequences from the genome are chosen that match 1) the GC% of the overall DNA sequence fragments analyzed and 2) their positional *k*-mer nucleotide biases, such as higher A or G at the +1 TSS (for k=1), or higher CA at position -1 (for k=2), etc.. DNA sequence motifs enriched at a given position in the input sequences are then identified by comparing against background sequences with matching positional and fragment-wide nucleotide content.

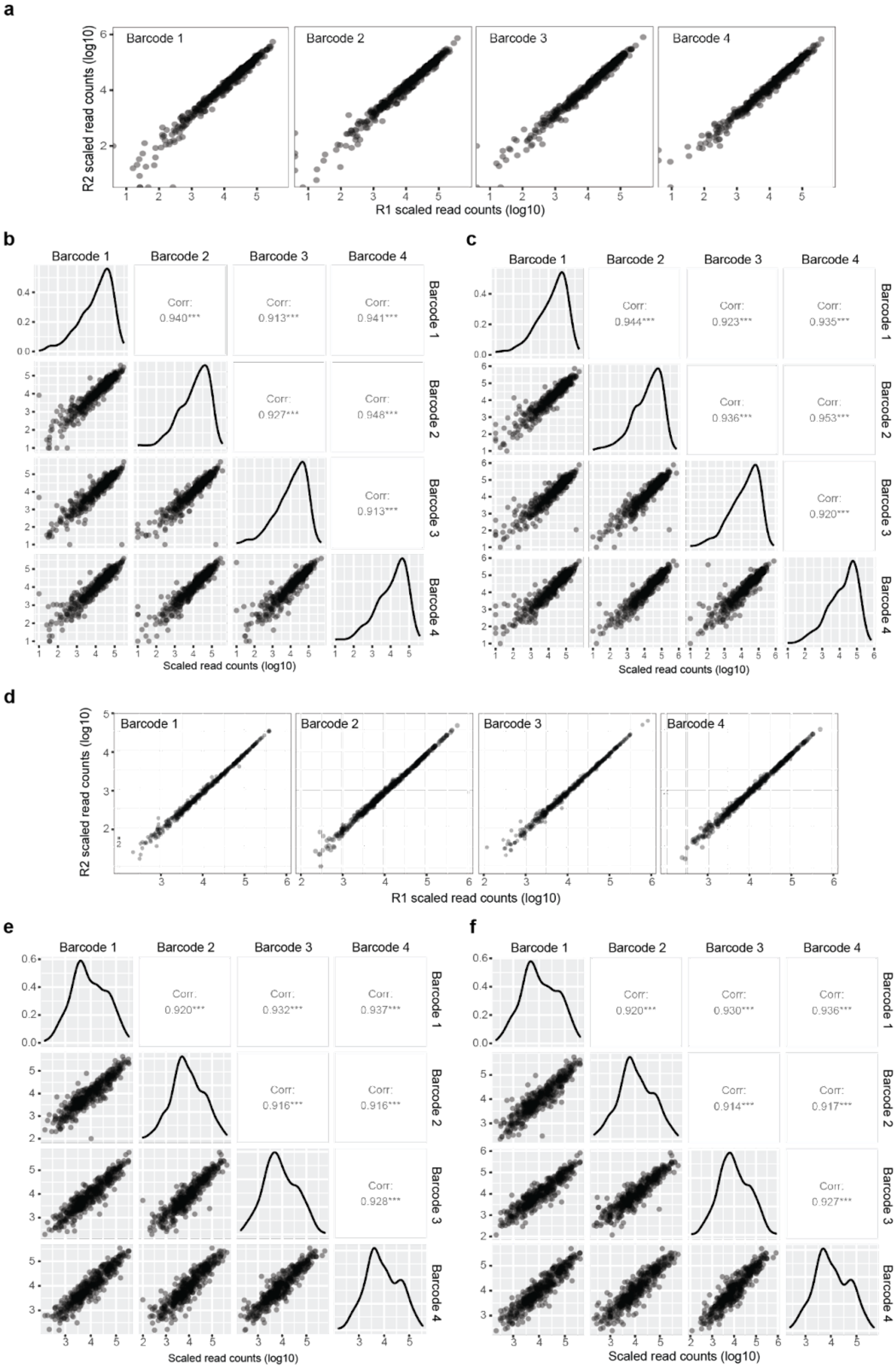
a
Barcode 1
Barcode 2
Barcode 3
Barcode 4
R2 scaled read counts (log10)
R1 scaled read counts (log10)
b
Corr: 0.940***
Corr: 0.913***
Corr: 0.941***
Corr: 0.927***
Corr: 0.948***
Corr: 0.913***
Scaled read counts (log10)
c
Corr: 0.944***
Corr: 0.923***
Corr: 0.935***
Corr: 0.936***
Corr: 0.953***
Corr: 0.920***
Scaled read counts (log10)
d
R2 scaled read counts (log10)
R1 scaled read counts (log10)
e
Corr: 0.920***
Corr: 0.932***
Corr: 0.937***
Corr: 0.916***
Corr: 0.916***
Corr: 0.928***
Scaled read counts (log10)
f
Corr: 0.920***
Corr: 0.930***
Corr: 0.936***
Corr: 0.914***
Corr: 0.917***
Corr: 0.927***
Scaled read counts (log10)

Supplementary Figure 6. Reproducibility of MPRA results across different downstream barcode sequences. Each synthetic DNA construct was assessed using four independent barcodes and MPRAs were additionally performed using two biological replicates. **a-c**, TF insertion TSS-MPRA (Fig.3) **a**, Scatterplot comparing biological replicate TSS activity levels across all four independent barcodes from the TF binding site sweep TSS-MPRA. **b**, Scatterplot comparing obtained measurements for a given DNA fragment across all four barcodes in replicate one and **c**, replicate two to assess the impact of the barcode sequence on TSS selection and transcription strength. **d-f**, TF sweep TSS-MPRA (Fig.4); **d**, Scatterplot comparing biological replicate TSS activity levels across all four independent barcodes from the TF binding site sweep TSS-MPRA. **e**, Scatterplot comparing obtained measurements for a given DNA fragment across all four barcodes in replicate one and **f**, replicate two to assess the impact of the barcode sequence on TSS selection and transcription strength.

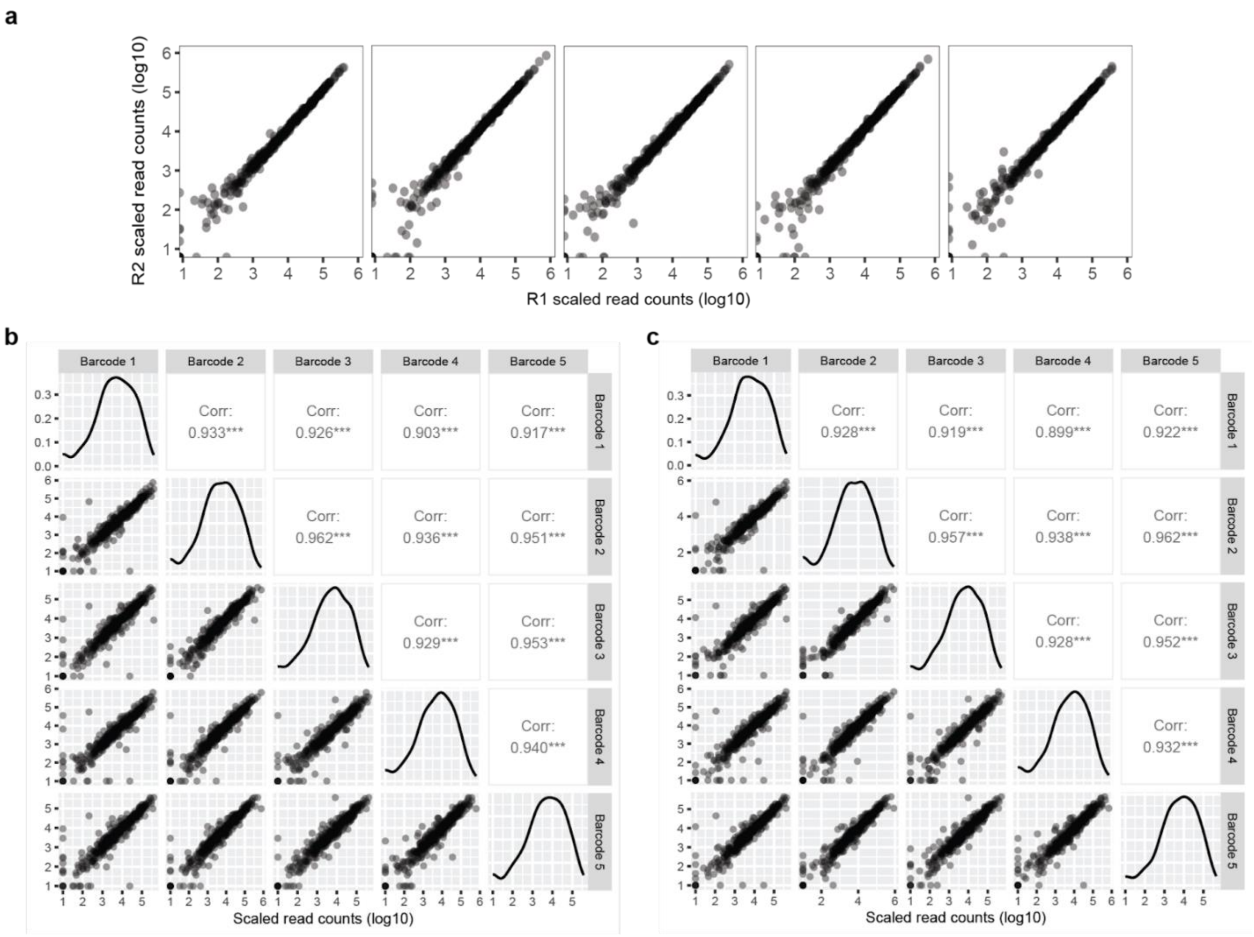

Supplementary Figure 7. Reproducibility of MPRA results across different downstream barcode sequences. Each synthetic DNA construct was assessed using four independent barcodes and MPRAs were additionally performed using two biological replicates. **a-c**, human TSR mutation TSS-MPRA (Fig.5) **a**, Scatterplot comparing biological replicate TSS activity levels across all four independent barcodes from the TF binding site sweep TSS-MPRA. **b**, Scatterplot comparing obtained measurements for a given DNA fragment across all four barcodes in replicate one and **c**, replicate two to assess the impact of the barcode sequence on TSS selection and transcription strength.

Supplementary References

1 Link, V. M. *et al.* Analysis of Genetically Diverse Macrophages Reveals Local and Domain-wide Mechanisms that Control Transcription Factor Binding and Function. *Cell* **173**, 1796-1809.e1717 (2018).

2 Shen, Z., Hoeksema, M. A., Ouyang, Z., Benner, C. & Glass, C. K. MAGGIE: leveraging genetic variation to identify DNA sequence motifs mediating transcription factor binding and function. *Bioinformatics* **36**, i84-i92 (2020).
