# Supplementary material for: Position-dependent function of human sequence-specific transcription factors
Source: Nature. 2024 Jul 17;631(8022):891–8. doi: 10.1038/s41586-024-07662-z (PMC11269187; doi:10.1038/s41586-024-07662-z)
Supplement: Supplementary file 2 — Reporting Summary [file 41586_2024_7662_MOESM2_ESM.pdf]

nature portfolio

Corresponding author(s): Benner, Christopher
Duttke, Sascha
Heinz, Sven

Last updated by author(s): Apr 26, 2024

Reporting Summary

Nature Portfolio wishes to improve the reproducibility of the work that we publish. This form provides structure for consistency and transparency in reporting. For further information on Nature Portfolio policies, see our Editorial Policies and the Editorial Policy Checklist.

Statistics

For all statistical analyses, confirm that the following items are present in the figure legend, table legend, main text, or Methods section.

n/a	Confirmed	
☐	☒	The exact sample size (*n*) for each experimental group/condition, given as a discrete number and unit of measurement
☐	☒	A statement on whether measurements were taken from distinct samples or whether the same sample was measured repeatedly
☐	☒	The statistical test(s) used AND whether they are one- or two-sided<br>*Only common tests should be described solely by name; describe more complex techniques in the Methods section.*
☐	☒	A description of all covariates tested
☐	☒	A description of any assumptions or corrections, such as tests of normality and adjustment for multiple comparisons
☐	☒	A full description of the statistical parameters including central tendency (e.g. means) or other basic estimates (e.g. regression coefficient) AND variation (e.g. standard deviation) or associated estimates of uncertainty (e.g. confidence intervals)
☐	☒	For null hypothesis testing, the test statistic (e.g. F, t, r) with confidence intervals, effect sizes, degrees of freedom and P value noted<br>*Give P values as exact values whenever suitable.*
☒	☐	For Bayesian analysis, information on the choice of priors and Markov chain Monte Carlo settings
☒	☐	For hierarchical and complex designs, identification of the appropriate level for tests and full reporting of outcomes
☐	☒	Estimates of effect sizes (e.g. Cohen's *d*, Pearson's *r*), indicating how they were calculated

Our web collection on statistics for biologists contains articles on many of the points above.

Software and code

Policy information about availability of computer code

Data collection	No specialized software was used for data collection

Data analysis

Code used to analyze data in this manuscript has been integrated into HOMER, or is available from the following repositories as described in the methods:
HOMER2 (HOMER v5) (http://homer.ucsd.edu/homer2)
MEIRLOP v0.0.16 (https://github.com/npdeloss/meirlop)
MEPP v0.0.1 (https://github.com/npdeloss/mepp)
MAGGIE v1.1.1 (https://github.com/zeyang-shen/maggie)

Additional Software:
cutadapt (v3.4)
STAR (v2.7.10a)
R (v4.2.2)
DESeq2 (v1.38.3)
bcftools (v1.6)
plink2 (v2.00a2.3LM)
TensorQTL (v1.0.3)
Fiji (V 1.53j)
Cluster 3.0 (v3.0)
Java TreeView (v1.1.6r4)
Excel (v16.83)
UCSC and IGV online Genome Browsers.

For manuscripts utilizing custom algorithms or software that are central to the research but not yet described in published literature, software must be made available to editors and reviewers. We strongly encourage code deposition in a community repository (e.g. GitHub). See the Nature Portfolio guidelines for submitting code & software for further information.

Data

Policy information about availability of data

All manuscripts must include a data availability statement. This statement should provide the following information, where applicable:
- Accession codes, unique identifiers, or web links for publicly available datasets
- A description of any restrictions on data availability
- For clinical datasets or third party data, please ensure that the statement adheres to our policy

All raw and processed data generated for this study can be accessed at NCBI Gene Expression Omnibus (GEO; https://www.ncbi.nlm.nih.gov/geo/) accession number GSE199431 (https://www.ncbi.nlm.nih.gov/geo/query/acc.cgi?acc=GSE199431). Previously published GEO and high-throughput sequencing datasets analyzed as part of this study include csRNA-seq data in C57Bl/6 mouse macrophages (GSE135498, https://www.ncbi.nlm.nih.gov/geo/query/acc.cgi?acc=GSE135498), NFY knockdown Start-seq data in mouse MEFs (GSE115110, https://www.ncbi.nlm.nih.gov/geo/query/acc.cgi?acc=GSE115110), PRO-cap data from 69 human lymphoblastoid cell lines (GSE110638, https://www.ncbi.nlm.nih.gov/geo/query/acc.cgi?acc=GSE110638), NRF1 ChIP-seq data from ENCODE in HepG2 (ENCSR853ADA, https://doi.org/doi:10.17989%2FENCSR853ADA) and K562 (ENCSR494TDU, https://doi.org/doi:10.17989%2FENCSR494TDU) cells. Genomes used for the analysis of sequencing data include Human: GRCh38/hg38 (https://hgdownload.soe.ucsc.edu/goldenPath/hg38/bigZips/hg38.fa.gz), Mouse(C57Bl/6): GRCm38/mm10 (https://hgdownload.soe.ucsc.edu/goldenPath/mm10/bigZips/mm10.fa.gz), Mouse(SPRET): GCA_001624865.1 (https://www.ncbi.nlm.nih.gov/datasets/genome/GCA_001624865.1/), and Green Monkey: Chlorocebus_sabeus 1.1/chlSab2 (https://hgdownload.soe.ucsc.edu/goldenPath/chlSab2/bigZips/chlSab2.fa.gz). Gene annotations were downloaded from GENCODE (Human v34, Mouse v25, https://www.gencodegenes.org/), and disease-risk variants from the GWAS Catalog mapping to hg38 were download from the UCSC Genome Browser (https://hgdownload.soe.ucsc.edu/goldenPath/hg38/database/gwasCatalog.txt.gz).
Liftover files for mapping between mouse strains were download from http://hgdownload.cse.ucsc.edu/goldenpath/mm10/liftOver/mm10ToGCA_001624865.1_SPRET_EiJ_v1.over.chain.gz (C57Bl/6/mm10 to SPRET) and https://hgdownload.soe.ucsc.edu/goldenPath/GCA_001624865.1_SPRET_EiJ_v1/liftOver/GCA_001624865.1_SPRET_EiJ_v1ToMm10.over.chain.gz (SPRET to C57Bl/6/mm10). Per-chromosome VCF files containing genotyping data for the samples analyzed in Kristjánsdóttir et al 63 were downloaded from the 1000 Genomes Project (ftp://ftp.1000genomes.ebi.ac.uk/vol1/ftp/data_collections/1000G_2504_high_coverage/working/20201028_3202_raw_GT_with_annot/).

Field-specific reporting

Please select the one below that is the best fit for your research. If you are not sure, read the appropriate sections before making your selection.

☒ Life sciences ☐ Behavioural & social sciences ☐ Ecological, evolutionary & environmental sciences

For a reference copy of the document with all sections, see nature.com/documents/nr-reporting-summary-flat.pdf

Life sciences study design

All studies must disclose on these points even when the disclosure is negative.

Sample size

No statistical methods were used to predetermine sample size. Nearly all assays performed in this study were profiled as biological replicates (n=2), starting from distinct biological material grown and treated as distinct samples. Resulting sequencing data was normalized and analyzed using R/DESeq2, and if more than 1% of features were considered differentially regulated using an FDR cutoff of 5% (or otherwise stated in the manuscript) we determined the experiment had a sufficient number of replicates. Please see Table S1 for an overview of samples collected and their replicate annotation.

Data exclusions

No data was excluded in the context of this study.

Replication	A minimum of duplicate experiments was performed for all experiments, and all attempts at replication were successful. In some experiments, additional replicates were part of the experimental design, such as the replicate inserts with differing barcodes in the TSS-MPRA experiments, which also replicated across the duplicates. For the ChIP-seq experiments, technical replicate experiments were performed with different antibodies on the same batch of cells. Replicability of the peak read count distributions across the genome between replicates, as well as the similarity of motif enrichment scores derived from peaks above the significance threshold that were obtained when using different antibodies on the same cells indicated a high level of replicability of the genome-wide transcription factor location analyses
Randomization	Randomization was not relevant to our study, and experiments were not randomized.
Blinding	No interventions that would require blinding to exclude bias were performed.<br>The investigators were not blinded to allocation during experiments and outcome assessment.

Reporting for specific materials, systems and methods

We require information from authors about some types of materials, experimental systems and methods used in many studies. Here, indicate whether each material, system or method listed is relevant to your study. If you are not sure if a list item applies to your research, read the appropriate section before selecting a response.

Materials & experimental systems

n/a	Involved in the study
☐	☒ Antibodies
☐	☒ Eukaryotic cell lines
☒	☐ Palaeontology and archaeology
☒	☐ Animals and other organisms
☒	☐ Human research participants
☒	☐ Clinical data
☒	☐ Dual use research of concern

Methods

n/a	Involved in the study
☐	☒ ChIP-seq
☒	☐ Flow cytometry
☒	☐ MRI-based neuroimaging

Antibodies

Antibodies used	anti YY1 (western, Santa Cruz, sc-7341 HRP YY1 (H-10)), anti YY1 (ChIP, ActiveMotif AB_2793763), anti NRF1 (Abcam , ab55744), anti β-Actin (Cell Signaling D6A8 - 8457S, Rabbit mAb #8457), and anti HA (Abcam, ab9110)<br><br>Antibody dilution for western blots:<br>anti-NRF1 (ab55744), 1:1000<br>anti-YY1 (sc-7341) 1:200<br>anti-beta actin (Cell Signaling D6A8 - 8457S, Rabbit mAb #8457) 1:2500<br><br>For ChIP-seq:<br>anti-HA (Abcam ab9110), rabbit pAb, 2 µg for 1x106 cells<br>anti-NRF1 (ab175932), rabbit mAb, 2 µg for 2.5x106 cells<br>anti-YY1 (Active Motif 61980, RRID: AB_2793763), rabbit pAb, 2 µg for 2.5x106 cells
Validation	Commercial validated monoclonal and polyclonal antibodies were further assessed by apparent molecular weight in western blot of the detected proteins across multiple cell lines (Supplementary Fig. 4a). Anti-NRF1 (ab55744) recognizes human NRF1 aa 201-285. anti-YY (sc-7341) was raised against full-length human YY1. The rabbit antibodies used for ChIP-seq were validated by ChIP-seq with additional mouse monoclonal antibodies that recognize the same antigens (anti-HA (Biolegend 901501), anti-NRF1 (Diagenode C15200013), anti-YY1 (Diagenode C15410345)), which resulted in near-identical ChIP-seq enrichment patterns.

Eukaryotic cell lines

Policy information about cell lines

Cell line source(s)	Cell lines used in this study were gifted from Aaron Carlin's lab (U2OS, Vero E6), Xiangdong Fu's lab (HepG2), and James Kadonaga's Lab (HEK293T) from University of California, San Diego.
Authentication	Cells lines were verified by the presence of cell-line specific DNA variants and by the phenotypic similarity of their genomics data to published resources (e.g. TSS locations identified in e.g. U2OS cells match open chromatin regions in U2OS cells identified by the ENCODE consortium).
Mycoplasma contamination	Cells were routinely tested for mycoplasma contamination. All tests were negative.
Commonly misidentified lines<br>(See ICLAC register)	No commonly misidentified cell lines were used in the study.

ChIP-seq

Data deposition

☒ Confirm that both raw and final processed data have been deposited in a public database such as GEO.

☒ Confirm that you have deposited or provided access to graph files (e.g. BED files) for the called peaks.

Data access links<br>*May remain private before publication.*	All raw and processed data generated for this study can be accessed at NCBI Gene Expression Omnibus (GEO; https://www.ncbi.nlm.nih.gov/geo/) accession number GSE199431.
Files in database submission	ChIP-seq peak file provided as a supplemental file: GSE199431_peaks.U2OS.dnNRF1.HA.bed.gz, GSE199431_peaks.U2OS.NRF1.bed.gz, GSE199431_peaks.U2OS.YY1.bed.gz
Genome browser session<br>(e.g. UCSC)	ChIP and csRNA-seq data are included for genomes hg38 and mm10:<br>https://genome.ucsc.edu/s/Cbenner/Reviewer%2DSession%2D240306%2DTfMotifGrammar

Methodology

Replicates	Only a single experiment was performed.
Sequencing depth	>10 million reads
Antibodies	anti YY1 (ChIP, ActiveMotif AB_2793763), anti NRF1 (Abcam , ab55744), and anti HA (Abcam, ab9110), which was used to target a dominant negative NRF1 protein tagged with HA.
Peak calling parameters	Peaks were found using HOMER's findPeaks program: findPeaks IP_Data/ -i Input_Data -style factor -o auto
Data quality	6,548 (NRF1), 9,454 (YY1) and 28,450 (dnNRF1) total peaks were identified, and the top de novo motif identifed using HOMER's motif analysis program for each experiment was a match for either the NRF1 or YY1 motif.
Software	HOMER v5.0 was used for the ChIP-seq analysis.
